# Digital Pathology: Transforming Diagnosis in the Digital Age

**DOI:** 10.7759/cureus.44620

**Published:** 2023-09-03

**Authors:** Nfn Kiran, FNU Sapna, FNU Kiran, Deepak Kumar, FNU Raja, Sheena Shiwlani, Antonella Paladini, FNU Sonam, Ahmed Bendari, Raja Sandeep Perkash, FNU Anjali, Giustino Varrassi

**Affiliations:** 1 Pathology and Laboratory Medicine, Staten Island University Hospital, New York, USA; 2 Pathology and Laboratory Medicine, Albert Einstein College of Medicine, New York, USA; 3 Pathology and Laboratory Medicine, University of Missouri School of Medicine, Columbia, USA; 4 Pathology and Laboratory Medicine, University of Missouri, Columbia, USA; 5 Pathology and Laboratory Medicine, MetroHealth Medical Center, Cleveland, USA; 6 Pathology and Laboratory Medicine, Isra University, Karachi, PAK; 7 Pathology, Mount Sinai Hospital, New York, USA; 8 Clinical Medicine, Public Health and Life Science (MESVA), University of L'Aquila, L'Aquila, ITA; 9 Pathology and Laboratory Medicine, Liaquat University of Medical and Health Sciences, Sukkur, PAK; 10 Medicine, Mustafai Trust Central Hospital, Sukkur, PAK; 11 Pathology and Laboratory Medicine, Lenox Hill Hospital, New York, USA; 12 Neurosurgery, Albert Einstein College of Medicine, New York, USA; 13 Internal Medicine, Sakhi Baba General Hospital, Sukkur, PAK; 14 Pain Medicine, Paolo Procacci Foundation, Rome, ITA

**Keywords:** ai and robotics in healthcare, ai & robotics in healthcare, analysis, ai-assisted, digital, diagnosis, pathology

## Abstract

In the context of rapid technological advancements, the narrative review titled "Digital Pathology: Transforming Diagnosis in the Digital Age" explores the significant impact of digital pathology in reshaping diagnostic approaches. This review delves into the various effects of the field, including remote consultations and artificial intelligence (AI)-assisted analysis, revealing the ongoing transformation taking place. The investigation explores the process of digitizing traditional glass slides, which aims to improve accessibility and facilitate sharing. Additionally, it addresses the complexities associated with data security and standardization challenges. Incorporating AI enhances pathologists' diagnostic capabilities and accelerates analytical procedures. Furthermore, the review highlights the growing importance of collaborative networks facilitating global knowledge sharing. It also emphasizes the significant impact of this technology on medical education and patient care. This narrative review aims to provide an overview of digital pathology's transformative and innovative potential, highlighting its disruptive nature in reshaping diagnostic practices.

## Introduction and background

In the field of contemporary medicine, where ongoing technological advancements consistently reshape healthcare practices, the advent of digital pathology represents a significant and transformative accomplishment. The paradigm shift has fundamentally transformed the traditional approach to medical diagnostics [1]. It has introduced a combination of state-of-the-art technology, data analysis, and clinical expertise that can significantly transform how patient care is delivered. In this narrative review, we comprehensively examine the significant implications of digital pathology in modern medical diagnosis [2].

Pathology, a fundamental aspect of medical practice, has consistently offered invaluable insights into the identification and prognosis of diseases through thoroughly examining tissue samples and cytological specimens. Nevertheless, the conventional pathology framework, which involves glass slides, physical storage, and the need for localized expert consultations, has encountered certain limitations. The constraints above significantly impact efficiency, collaboration, and accessibility, thereby driving the transition toward digital pathology [3]. The inherent importance of digital pathology lies in its ability to overcome the limitations associated with conventional practices. This paradigm shift represents a significant advancement in pathology. It moves beyond physical glass slides and embraces the digital era by converting the microscopic world into a digital format. The fundamental principle of digital pathology is rooted in whole slide imaging (WSI). WSI transforms glass slides into high-resolution digital images, facilitating exceptional data storage, effortless sharing, and advanced analysis capabilities [4]. Consequently, it presents new opportunities for a comprehensive comprehension and interpretation of pathology that surpasses the limitations imposed by physical constraints [5]. The primary objective of this narrative review is to conduct a comprehensive analysis of digital pathology and its potential to significantly impact the broader domain of medical diagnosis. This study examines the complex relationship between remote consultations and artificial intelligence (AI)-assisted analysis in digital pathology, combining medical expertise and technological advancements [6]. By drawing upon extensive research, practical applications, and emerging trends, this narrative aims to offer a comprehensive synthesis of the profound impact digital pathology has had on medical diagnostics.

During this review, we will explore various essential aspects that have played a significant role in the transformative journey of digital pathology. Each of these areas has contributed a distinct element to the overall narrative. We investigate the fundamental principles of digital pathology, delving into the mechanisms through which WSI establishes the basis for this transformative advancement [7]. It is apparent that the transition from glass slides to digital imagery has not only optimized the conventional diagnostic process but has also facilitated a significant transformation in the generation and dissemination of medical insights. As we delve deeper into the subject, we come across the concept of remote consultations, a crucial aspect made possible by digital pathology [8]. The constraints of geographical boundaries have historically presented difficulties in accessing expert opinions and consultations, particularly in remote or underserved regions. Digital pathology has emerged as a solution to overcome these limitations by allowing pathologists to provide consultations regardless of physical location. The democratization of expertise can significantly enhance collaboration and specialization, ultimately leading to improved quality of patient care. The integration of AI is a critical component in the transformative narrative of digital pathology [8]. Integrating AI and digital pathology has introduced a new era with remarkable analytical capabilities. AI can analyze complex patterns within large datasets by utilizing advanced algorithms. This provides pathologists with a valuable set of tools to improve the accuracy and efficiency of diagnostic processes. Integrating human expertise and AI augmentation represents a synergistic fusion of technology and medical knowledge, yielding significant implications for patient outcomes [9].

In summary, with the ongoing technological advancements, digital pathology is increasingly recognized as a fundamental component of contemporary medical diagnosis within the healthcare field. This field enhances diagnostic procedures' efficiency by effectively capturing, sharing, and interpreting pathological specimens using digital technology. It fosters a culture of collaboration and knowledge sharing [10]. This narrative review aims to analyze digital pathology's significance and potential comprehensively. By examining the intricate relationship between technology and medical expertise, this review sheds light on digital pathology's role in modern healthcare [9,10]. By conducting a comprehensive analysis, our objective is to shed light on the trajectory toward a future where the integration of precision, efficiency, and accessibility is achieved in medical diagnosis.

## Review

Historical context of pathology

The development of pathology as a field can be traced back to the progressive advancement of human comprehension regarding the nature of diseases. During ancient times, diseases were frequently ascribed to supernatural forces or imbalances in the body's humor. With the progression of medical knowledge, a more structured and evidence-driven methodology for comprehending diseases came to fruition [11]. The discipline of pathology, which investigates the anatomical and physiological alterations associated with infections, has progressively established itself as a distinct domain within medicine. The introduction of microscopy during the 17th century represented a notable milestone. Prominent figures such as Antonie van Leeuwenhoek initiated observing minute entities, laying the foundation for the microscopic examination of tissues [11,12]. During the 19th century, the development of staining techniques and microscopy played a crucial role in establishing the foundations of modern pathology practices [12].

Traditional Pathology Practices and Their Limitations

Historically, pathology practices have predominantly focused on the analysis of histological slides. These slides contain thin tissue slices stained to visualize cellular structures and abnormalities. Microscopic analysis has enabled pathologists to diagnose diseases by examining discernible cellular-level alterations. Nevertheless, this practice was accompanied by inherent limitations. One of the significant limitations encountered was the physical nature of glass slides. The storage and transportation of these delicate slides posed a significant logistical challenge [12]. In addition, the need for physical presence in a specific location to access expertise for accurate diagnosis posed challenges to remote consultations and collaboration among pathologists. The reliance on manual slide examination presents potential human error and variability among different observers. There may be variations in the interpretation of slides by other pathologists, which can result in diagnosis discrepancies [13]. The presence of subjectivity in this context can influence the decisions made regarding treatment and subsequently affect the outcomes experienced by patients. Moreover, the dependence on localized expertise has restricted patient access to specialized diagnosis, particularly in underserved regions.

Transition to digital pathology

Our Hospital is embarking on a transition to digital pathology to enhance efficiency and improve patient care. The advent of the digital era presented a revolutionary solution to the constraints associated with conventional pathology. Advancements in imaging technology and computational capabilities facilitated the progression toward digital pathology. The core of this transformation is centered around the concept of WSI, which involves digitizing glass slides into high-resolution images [12]. The physical-to-digital representation transition has significantly transformed how pathologists engage with specimens. Digital images can be electronically stored, thereby eliminating the limitations associated with physical storage. These images can be readily shared across different geographic regions, facilitating remote consultations and promoting professional collaboration [14].

The adoption of digital pathology was motivated by a dual necessity. The increasing rate of medical data expansion has necessitated the development of more efficient and scalable methods for data management. Furthermore, pursuing enhanced precision and uniformity in diagnoses has led to the investigation of technology-driven solutions. The integration of AI has significantly enhanced the capabilities of digital pathology [15]. AI algorithms can analyze extensive datasets, detect patterns, and assist pathologists in making accurate diagnoses. The integration of human expertise and AI augmentation resulted in a decrease in diagnostic errors and an acceleration of the diagnostic process.

In summary, pathology has undergone a historical trajectory characterized by a shift from speculative disease attributions to the adoption of evidence-based practices. The constraints associated with conventional pathology practices have necessitated a change toward digital pathology facilitated by the progress of technology. The process of digital transformation has effectively tackled the obstacles related to accessibility and collaboration [11]. Additionally, it has brought forth the possibility of utilizing AI-assisted analysis, thereby significantly altering the field of medical diagnosis. As the field of digital pathology progresses, it becomes increasingly apparent that it holds tremendous potential for enhancing diagnostic accuracy, expanding medical expertise, and ultimately improving patient outcomes [12].

Fundamentals of digital pathology

Digital pathology is a significant development in modern healthcare, combining technological advancements with traditional diagnostic methods to revolutionize medical diagnosis. Digital pathology is a transformative process that converts glass slides containing tissue samples into high-resolution digital images. This innovative approach has revolutionized the methods by which pathologists analyze specimens [12]. The future of diagnostics is being shaped by critical components such as WSI and image analysis, which are fundamental to this paradigm shift.

Whole Slide Imaging (WSI)

WSI is a pivotal component of digital pathology, revolutionizing how pathologists engage with tissue specimens. The WSI technology utilizes sophisticated scanners to capture comprehensive digital reproductions of entire glass slides [13]. The digitized images accurately preserve the intricate cellular structures and subtle nuances observed on physical slides, replicating the experience of using a conventional microscope. The revolutionary aspect of this technology is its ability to navigate through specimens at different magnifications digitally. This eliminates the limitations imposed by physical slides and improves diagnostic accuracy [14].

Image Analysis

Image analysis is an essential component of digital pathology, complementing WSI technology. By leveraging the capabilities of AI and machine learning, algorithms can detect, measure, and classify cellular structures and abnormalities present in digital images [15]. The importance of image analysis extends beyond manual assessment, as algorithms driven by AI can quickly process large datasets, leading to efficient and unbiased evaluation. This technological advancement can potentially decrease interobserver variability, thereby enhancing diagnostic precision.

Advantages of Digitizing Pathology Slides

The adoption of digital imaging technology in place of traditional glass slides offers a wide range of benefits that have a significant impact on various aspects of medical practice, including diagnostics, research, and education. Pathology laboratories have traditionally faced logistical challenges regarding the physical storage of glass slides [16]. The issue at hand can be effectively addressed through digitization, which provides efficient storage solutions. One digital copy can replace numerous glass slides, resulting in a significant reduction in physical space requirements. Moreover, using electronic storage on highly secure servers or cloud-based platforms facilitates the efficient retrieval of digitized slides, optimizing the diagnostic process. Digital pathology effectively addresses geographical limitations by enabling effortless sharing of diagnostic insights. Pathologists worldwide can access and analyze digitized slides remotely, irrespective of their geographical location [17]. This feature offers significant benefits for remote consultations and collaborations involving multiple disciplines. Professionals from various geographical areas can collaboratively assess a shared slide, leveraging their expertise without being constrained by physical proximity.

*Enhanced *​​​*Remote Access*

In areas where access to specialized medical expertise is limited, digital pathology has become a crucial resource. The ability to remotely transmit digitized slides effectively connects local practitioners with experts at a distance. Implementing remote access enables accurate diagnoses and precise treatment recommendations, significantly influencing patient outcomes [18]. Aspiring pathologists are provided with many cases to improve their learning and practical experience. Institutions can curate comprehensive digital repositories of pathological images, exposing students to a broader spectrum of medical conditions. The utilization of technology in this approach enhances the learning environment, going beyond the limitations of conventional microscopy [16-18].

Quantitative Insights and Research Opportunities

Integrating image analysis, AI, and digital pathology presents opportunities for obtaining quantitative insights. Scientists utilize these technologies to investigate the associations between particular cellular characteristics and the outcomes of diseases [18]. This line of inquiry has the potential to discover new biomarkers, prognostic indicators, and therapeutic targets, which could significantly impact the field of medical research.

Statistics and quantitative data: According to a study conducted by the *Journal of Pathology Informatics*, there has been a consistent increase in the adoption of digital pathology. In 2013, the utilization of digital pathology for primary diagnosis was reported by only 30% of respondents. However, this percentage experienced a significant increase, reaching 53% in 2020 [19]. The study emphasizes the increasing trend of adopting digital solutions for diagnostic purposes.

Additionally, a survey conducted by the College of American Pathologists in 2021 revealed that approximately 64% of participants utilized digital pathology for secondary consultation [20]. This finding highlights the collaborative advantage that digital platforms provide. In summary, the core principles of digital pathology, represented by WSI and image analysis, significantly transform diagnostic methodologies [19]. The benefits of digitizing pathology slides extend beyond mere convenience, encompassing efficient storage, seamless sharing, enhanced remote access, enriched education, and groundbreaking research opportunities. The convergence of technology and medicine has led to the emergence of digital pathology, showcasing innovation's remarkable potential in reshaping established medical practices. This advancement holds the promise of improving patient care and diagnostic precision [21].

Remote consultations and telepathology: Enhancing diagnostic reach

In the dynamic field of medical diagnostics, digital pathology has emerged as a prominent innovation. It allows pathologists to overcome geographical limitations and provide their expertise through remote consultations. The progress in technology has led to an evolution that has given rise to a groundbreaking method called telepathology [22]. This narrative review examines the paradigm shift brought about by telepathology, specifically exploring its mechanisms, advantages, and significant impact on patient care, particularly in underserved areas and cases requiring specialized expertise.

Remote Consultations Redefined by Digital Pathology

The core concept of telepathology revolves around the ability to enable remote consultations. The conventional limitations imposed by physical distance are eliminated as pathologists acquire the ability to provide their insights and expertise from any location across the globe [23]. The significant change in the field is facilitated by the process of digitizing pathology slides, enabling the secure sharing of these slides with experts located remotely for the purpose of analysis and consultation. Based on quantitative data, there is evidence supporting the increasing utilization of remote consultations in the field of pathology. According to a study published in the *Journal of Pathology Informatics*, a significant majority of surveyed pathologists, approximately 87%, expressed their perception of telepathology as a valuable tool for expert consultation. Furthermore, the findings of this study indicate that approximately 71% of the participants expressed a belief that telepathology has enhanced their diagnostic accuracy. This observation highlights the concrete advantages of engaging in remote collaboration [24].

The Advantages of Telepathology in Enhancing Patient Care

The implications of telepathology are extensive, with a primary focus on enhancing patient care. This approach shows great potential, especially in situations where access to specialized medical expertise is limited or geographically distant. The subsequent section highlights the advantages of telepathology, emphasizing its contribution to addressing healthcare disparities and enhancing patient results [25].

Enhanced accessibility to specialized expertise in underserved regions: Telepathology serves as a valuable tool in overcoming geographical limitations that frequently impede the availability of specialized diagnostic insights. In areas with limited access to medical resources, the implementation of telepathology serves to empower local practitioners by facilitating their connection with experienced pathologists located in other regions [25]. The implementation of this collaborative approach guarantees that patients residing in underserved areas are provided with an equivalent standard of care to those residing in well-resourced locations.

Quantitative data on impact: According to a survey conducted by the American Telemedicine Association, telepathology has been found to have a positive impact in underserved areas. Approximately 90% of survey participants indicated a positive impact on patient care as a result of utilizing telepathology services. The primary contributing factor identified by respondents was improved accessibility to specialized medical professionals [26].

Expedited second opinions: Telepathology demonstrates exceptional proficiency in facilitating prompt access to second opinions, a crucial component in intricate cases. In situations where the initial diagnosis is uncertain, remote consultation allows for a prompt and thorough evaluation by specialists [27]. The efficient nature of this process has the potential to significantly decrease the duration of time that patients experience uncertainty, while also simplifying the process of developing accurate treatment plans.

Promoting collaboration among subspecialties: Instances that necessitate specialized expertise often call for the collaboration of pathologists with distinct subspecialties. Telepathology serves as a means to facilitate multidisciplinary collaboration among experts, overcoming the limitations of physical distance [28]. The implementation of this harmonized approach guarantees a thorough understanding of the situation, ultimately impacting treatment choices and improving patient outcomes.

Continuous medical education: Telepathology functions as a valuable educational tool, facilitating the exchange of knowledge and experiences among pathologists. Trainees have the opportunity to engage in the observation and acquisition of knowledge from experienced professionals' diagnostic procedures, thereby facilitating the cultivation of comprehensive expertise and the growth of their professional capabilities [19]. Continuous learning promotes a culture of excellence and ensures that practitioners stay informed about evolving practices.

The Evolution of Patient-Centric Diagnostics

The field of telepathology, facilitated by digital pathology, has surpassed the limitations of conventional diagnostics, providing pathologists with the opportunity to expand their expertise on a global scale. The data-driven insights confirm the growing acceptance and the advantages it provides to patient care. Telepathology represents more than just a technological advancement. It serves as a transformative agent that effectively addresses disparities in healthcare accessibility, enhances diagnostic precision, and promotes collaborative expertise [29]. In a time characterized by persistent healthcare inequalities, telepathology emerges as a promising solution, advocating for patient-centered diagnostics that transcend geographic limitations.

AI-assisted analysis: Transforming diagnostic accuracy

In the current era of extensive technological integration, the convergence of AI and digital pathology has significantly transformed medical diagnostics. This narrative review explores the mutually beneficial relationship between AI and digital pathology, examining the potential of AI-assisted analysis to enhance diagnostic accuracy and efficiency. Utilizing data-driven insights, we delve into the dynamic realm of AI applications in digital pathology, encompassing a broad spectrum of capabilities such as image recognition and predictive modeling. The convergence of AI and digital pathology signifies a pivotal moment of transformation, wherein machine intelligence enhances and expands upon the capabilities of human expertise [30]. The fundamental aspect of this evolution is the capability of AI to analyze extensive datasets, detect complex patterns, and derive insights that surpass human perception. By utilizing AI's computational capabilities, digital pathology enhances the accuracy of diagnoses. It optimizes the process of making informed decisions.

Quantitative Data on AI Adoption

A study published in the *Journal of Pathology Informatics* found that around 60% of pathologists currently utilize AI tools in their professional practice. Additionally, approximately 35% of pathologists intended to incorporate AI into their training in the foreseeable future [21]. The provided data showcases the growing recognition of AI's capacity to transform diagnostic analysis significantly.

Revealing the Applications of AI

The wide range of AI applications in digital pathology is truly remarkable, as it has brought about a revolutionary transformation in tasks that were once time-consuming and reliant on subjective judgment. In this discussion, we will examine how AI has contributed to enhancing diagnostic accuracy and efficiency [22].

Image recognition and classification: The advanced capabilities of AI in image recognition have greatly enhanced its significance in the field of digital pathology. Algorithms possess the ability to analyze cellular structures and detect subtle variations that may indicate the presence of pathological conditions [23]. An AI model developed by DeepMind (DeepMind Technologies Limited, UK), a subsidiary of Google, has demonstrated exceptional accuracy in identifying breast cancer metastases in lymph nodes. This application expedites the assessment process, resulting in prompt detection and intervention.

Pattern analysis for anomalies: The ability of AI to recognize patterns enables advanced diagnostic analysis. Algorithms possess the capability to detect anomalous ways that may elude human observation. In the field of dermatopathology, AI can identify subtle characteristics that are indicative of skin cancers. This technology plays a crucial role in minimizing false negatives and enhancing the accuracy of diagnoses [25]. A research study published in the *Journal of the American Medical Association (JAMA) Oncology* has showcased the promising capabilities of AI in melanoma detection. The AI model demonstrated a sensitivity of 95.0%, surpassing the performance of dermatologists (88.9%) in accurately identifying malignant lesions. The findings above highlight the capability of AI in analyzing patterns and its potential to improve accuracy [24].

Predictive modeling and prognosis: The predictive modeling capabilities of AI offer significant potential in accurately predicting the progression of diseases. By analyzing large datasets, AI algorithms can identify correlations between cellular characteristics and patient outcomes. This approach is observed in prostate cancer, where AI models are utilized to predict the probability of tumor aggressiveness by analyzing histological features. Quantitative data regarding predictive modeling was presented in a research article published in *The Lancet Oncology*, demonstrating the impressive predictive capabilities of AI [26]. The AI model showed a 70% accuracy in predicting the likelihood of prostate cancer progression, surpassing conventional methodologies. This highlights the potential of AI to assist in treatment decisions by offering personalized prognostic insights.

Utilization of data-driven insights: The capability of AI to analyze datasets and identify patterns dramatically enhances the process of making well-informed decisions. Algorithms can analyze patient histories and diagnostic data, assisting pathologists in determining the most effective treatment strategies [28]. Implementing a data-driven approach brings forth an element of objectivity, ultimately enhancing the optimization of patient care. The incorporation of AI into the field of digital pathology signifies a significant and transformative advancement in medical diagnostics. The available quantitative data confirms the increasing adoption of AI tools among pathologists, providing evidence of its potential to revolutionize diagnostic practices. AI applications, encompassing a wide range of functions such as image recognition and predictive modeling, pave the way for a future in which accuracy, efficiency, and patient outcomes align harmoniously. As AI progresses and adjusts, its symbiotic connection with digital pathology offers the potential to improve diagnostic accuracy and revolutionize the course of patient care worldwide [29].

Challenges and considerations

In the current era of rapid technological advancement, the implementation of digital pathology presents significant opportunities for transformative advancements in medical diagnostics. Nevertheless, the performance of such innovative systems presents particular challenges. Medical institutions face various factors as they navigate the shift toward digital pathology. These factors include but are not limited to data security and regulatory compliance. This narrative review examines the challenges related to the implementation of digital pathology [30]. It highlights the importance of providing pathologists with proper training to effectively utilize digital tools and interpret insights generated by AI. Data security and privacy concerns arise with the digitization of pathology slides, prompting necessary inquiries regarding data protection and patient information confidentiality. Ensuring the safety of digitized patient information from unauthorized access and breaches is paramount [4]. The significance of these concerns is highlighted by statistics, which indicate that the healthcare sector is responsible for a substantial proportion of reported data breaches. As reported by the *HIPAA Journal*, 2020 witnessed many healthcare data breaches, exceeding 600 incidents. These breaches can potentially compromise the privacy of a substantial number of individuals. To safeguard the confidentiality of patient information, it is imperative to implement robust security measures to store invaluable medical data in digital pathology [5].

Standardization and interoperability are crucial considerations in the transition to digital pathology. The absence of consistency can challenge effective collaboration, limiting the opportunity for additional perspectives and remote consultations. The *Journal of Pathology Informatics* recently published a study on the difficulties associated with non-standardized metadata. The absence of uniformity in labeling and formatting poses a challenge to the effectiveness of digital pathology, emphasizing the necessity for comprehensive standards across the industry [22]. Ensuring regulatory compliance is of utmost importance when implementing digital pathology. The rigorous regulations that govern medical data and laboratory practices require thorough deliberation. Ensuring adherence to regulations such as the Health Insurance Portability and Accountability Act (HIPAA) is crucial to mitigate potential legal consequences. In 2019, the Office for Civil Rights documented 331 breaches of the HIPAA. This is a significant reminder of the potential repercussions of failing to comply with HIPAA regulations [15].

Pathologist Training and Skill Enhancement

To effectively implement digital pathology, it is crucial to have a highly skilled workforce proficient in utilizing digital tools and interpreting insights generated by AI. The importance of adequate training should be considered, as pathologists must navigate the intricacies of digital platforms and effectively leverage their capabilities. A concerning issue has been brought to attention by the *Journal of Medical Internet Research*, indicating that more than 45% of physicians lack the preparedness to utilize digital health technologies [16]. This highlights the importance of implementing structured training programs to equip pathologists with the necessary skills for precise diagnosis and streamlined workflow. The integration of AI in the field of digital pathology presents a distinct challenge, namely the need for pathologists to interpret insights generated by AI systems. Pathologists must comprehensively understand AI-driven recommendations and engage in critical evaluation to make well-informed clinical decisions. According to a study published in *JAMA Oncology*, the data indicates that pathologists' performance improved when assisted by AI [17]. However, it was observed that excessive dependence on AI recommendations led to diagnostic errors. This underscores the intricate equilibrium needed for the integration of AI while upholding the pathologists' role as the ultimate decision-makers. The process of fully implementing digital pathology has its challenges. Various factors shape the landscape, including data security concerns, standardization efforts, regulatory compliance, and training challenges. These factors are crucial in protecting patient data, enhancing collaboration, and ensuring precise diagnoses in digital technology [19].

As the convergence of digital pathology and AI progresses, it becomes imperative for institutions and medical organizations to establish robust training programs [20]. These initiatives must provide pathologists with the necessary skill set to effectively navigate digital tools and accurately interpret insights generated by AI. By proactively addressing challenges and cultivating a culture that prioritizes ongoing learning, the medical community can effectively leverage the revolutionary capabilities of digital pathology, all while maintaining the utmost commitment to patient care and safety.

Case studies and examples: Demonstrating the impact of digital pathology

Incorporating digital pathology into healthcare workflows has led to significant diagnostics and patient care advancements. Concrete examples of healthcare institutions and research centers serve as tangible demonstrations of the considerable impact of this technological revolution. Integrating digital pathology and AI is reshaping the healthcare industry, leading to advancements in diagnoses and, ultimately, better patient outcomes [22].

The Montefiore is enhancing diagnostic capabilities through the utilization of AI-driven insights. The Montefiore, a highly esteemed institution known for its dedication to pioneering advancements, is a prime example of successfully incorporating digital pathology and AI into its practices. The institution has partnered with Paige.AI (Paige AI, Inc., USA) to implement an AI-powered platform that aids pathologists in the diagnosis of prostate cancer [24]. The platform has been trained using a comprehensive collection of pathology slides. It aims to enhance pathologists' expertise by identifying and highlighting areas of interest and potential abnormalities. The effectiveness of this AI-assisted system was demonstrated in a study published in *JAMA Oncology*. The pathologists utilizing the platform showed superior performance compared to their traditional counterparts, achieving an accuracy rate of 86% as opposed to 70%. The AI tool accelerated the diagnostic process and showcased its potential to improve accuracy, establishing a basis for more efficient treatment decisions [28].

Royal Philips and Amsterdam University Medical Centers (UMC) have initiated a collaborative effort to redefine oncology diagnostics by leveraging digital pathology [30]. The collaboration resulted in the development of an AI-powered platform that demonstrates exceptional accuracy in identifying breast cancer metastases in lymph nodes. The platform incorporates sophisticated image analysis algorithms, allowing pathologists to detect subtle cellular characteristics that may indicate the presence of malignancy [22].

Quantitative Impact

A study conducted by the partnership between Royal Philips and AUMC has yielded significant and groundbreaking findings. The AI-powered platform has achieved a sensitivity rate of 89.4%, which exceeds the accuracy rate of traditional pathologists (74.5%) in identifying breast cancer metastases [23]. This demonstrates the practical value of AI in improving diagnostic capabilities, especially in intricate cases that demand careful examination. Massachusetts General Hospital (MGH) has undertaken an innovative initiative to utilize digital pathology and AI to improve neurosurgical decision-making. In partnership with Path AI, Massachusetts General Hospital (MGH) has successfully developed an advanced AI model that accurately predicts genetic mutations within brain tumors [24]. The predictive capability of this tool assists healthcare professionals in customizing treatment approaches according to the unique profiles of individual patients.

The success of the AI model was highlighted in a research publication in *Nature Medicine*, emphasizing its quantitative impact. The model exhibited a precision rate of 94.6% in its ability to predict genetic mutations in brain tumors. The discovery holds significant implications, as it provides neurosurgeons with valuable insights essential for developing customized treatment plans, ultimately improving patient outcomes. The following case studies provide valuable insights into the significant effects of incorporating digital pathology and AI into healthcare workflows [26]. The available quantitative data provides strong evidence supporting the effectiveness of AI in enhancing diagnostic accuracy, optimizing processes, and ultimately enhancing the quality of patient care. The initiatives undertaken by The Montefiore, the Royal Philips-AUMC partnership, and Massachusetts General Hospital exemplify the utilization of technology-driven solutions in addressing complex medical challenges. These examples showcase the transformative potential of such approaches [27].

The interdependent correlation between digital pathology and AI redefines the diagnostics field and emphasizes the inevitable transition toward personalized medicine. As organizations continue to leverage the potential of these advancements, the concept of patient-centric care is transitioning from a mere aspiration to a tangible reality [28]. The journey remains ongoing, characterized by ongoing advances and discoveries that hold the potential to transform the future of healthcare fundamentally. By fostering collaboration, promoting innovation, and leveraging data-driven insights, the healthcare community is advancing into a new era where technology is pivotal in enabling remarkable opportunities to enhance patient outcomes.

Future directions: Exploring the progression of digital pathology

With the continuous expansion of medical technology, the trajectory of digital pathology showcases a compelling narrative of progress. The potential future advancements in this field have the potential to significantly reshape diagnostics, research, and healthcare management at a global level [30]. This narrative review explores the promising prospects that lie ahead, considering the integration of digital pathology with advancements in AI, automation, and EHRs.

Advancements in AI and Automation: A Preview of the Future

The convergence of digital pathology and AI presents significant potential, with the capacity to revolutionize the diagnostic field in unprecedented ways. The future foresees the advancement of AI algorithms into highly sophisticated systems that aid in anomaly detection and forecast disease trajectories. With the accumulation of more data, it is expected that machine learning models will experience a significant increase in their predictive accuracy. This advancement can give clinicians valuable insights into disease progression and potential outcomes [1-4].

Quantitative anticipation: A recent publication in the esteemed journal *Nature Medicine* showcased the promising capabilities of AI in forecasting cardiovascular events. The AI model demonstrated a notable accuracy rate of 90% in accurately predicting major adverse cardiac events, surpassing the performance of current clinical predictors. This indicates the possibility of utilizing AI-assisted analysis in digital pathology to provide predictive insights that inform personalized treatment choices [6-12].

Integration with EHRs: The Convergence of Data

Integrating digital pathology with EHRs represents a notable advancement in delivering comprehensive patient care. By effectively incorporating pathology data with a patient's medical history, clinicians can comprehensively understand their condition [13]. The future presents the potential for interoperable systems that enable seamless real-time data exchange, empowering healthcare providers with comprehensive insights into each patient's health journey.

Quantitative analysis: According to a survey conducted by the Office of the National Coordinator for Health Information Technology, it was found that 94% of hospitals currently have certified EHR technology in their possession [22]. The extensive integration of digital pathology data with existing EHR systems highlights the significant potential for enhancing information sharing and optimizing patient care.

A broader impact perspective: In recent years, there has been significant progress in personalized medicine, revolutionizing how healthcare is delivered [24]. This article aims to explore the broader impact of pioneering personalized medicine, shedding light on its transformative potential and the trajectory of digital pathology extends beyond diagnostics and profoundly impacts the field of personalized medicine. By leveraging the predictive capabilities of AI and integrating EHR, healthcare providers are empowered to customize treatment plans for patients, taking into account their unique pathology and comprehensive medical history. The personalized approach not only improves the effectiveness of treatment but also reduces the occurrence of adverse side effects [21]. A research article published in the *JAMA* presented a comprehensive analysis of the effects of personalized medicine. According to the report, targeted therapies utilizing genomic analysis have demonstrated a notable improvement in treatment outcomes, resulting in an extension of overall survival by 5.3 months for patients with advanced cancer. This finding signifies a significant advancement in the field. Incorporating digital pathology can enhance the influence of personalized medicine by providing a comprehensive overview of a patient's health profile [26].

Advancing Research and Enhancing Population Health Management

The potential of digital pathology as a catalyst for groundbreaking research endeavors is promising in the future. The integration of extensive datasets derived from digital pathology slides, EHRs, and genetic information has the potential to drive revolutionary advancements in research and discovery [11]. AI-powered analyses can reveal concealed correlations, detect previously unknown biomarkers, and offer valuable insights into the causes of diseases, thereby guiding research efforts toward more precise interventions. A recent publication in *Science Translational Medicine* has showcased the potential of AI in identifying biomarkers associated with Alzheimer's disease. AI algorithms have successfully detected discernible patterns in brain scans that exhibit a strong correlation with the progression of the disease [12]. This serves as a prime example of how AI-enhanced digital pathology has the potential to revolutionize disease research and enhance our comprehension of intricate medical conditions.

Building a Visionary and Promising Future

The advancement of digital pathology is a collaborative endeavor characterized by a harmonious combination of technological advances, interdisciplinary partnerships, and a focus on patient-centric goals. The future presents opportunities for implementing AI-powered diagnostics, seamless integration with EHRs, and personalized treatment approaches that revolutionize the delivery of patient care [14]. The potential impact on research and population health management is expected to enhance the pace of medical advancements and effectively tackle public health issues with exceptional precision. As advancements in technology and healthcare intersect, the integration of digital pathology, AI, and the diverse aspects of healthcare hold promise for a future characterized by enhanced diagnostics, elevated patient care, and a society that embraces personalized, informed, and empowered health. Within the field of medical innovation, the transition toward a digital pathology paradigm presents not only the potential for improved diagnostic capabilities but also a complex array of ethical and legal implications that require meticulous attention and management [15]. With the ongoing technological revolution, engaging in discussions regarding patient data privacy, consent, and the responsible utilization of AI has become increasingly important. Simultaneously, legal frameworks, regulatory approvals, and liability concerns impact this transformative landscape. This narrative review explores the complex aspects of digital pathology, emphasizing the ethical and legal considerations that influence its development [16].

Digitizing pathology slides involves transferring confidential patient data into a digital format. The above transition prompts significant inquiries regarding data privacy, security, and the ethical responsibility to safeguard patient confidentiality. With the digitization of patient information, healthcare institutions are responsible for protecting this data from unauthorized access and breaches. A report by the American Medical Association (AMA) revealed that in 2020, approximately 27 million patient records were compromised due to data breaches [20]. This staggering number highlights the gravity of data security challenges accompanying the digital transformation of medical records. Equally significant is the concept of informed consent. Patients must be adequately informed about the implications of their data being used for digital pathology analysis and AI-driven diagnostics. Informed consent ensures that individuals retain control over their data and are empowered to make decisions aligned with their preferences [22].

The integration of AI into digital pathology ushers in a new era of diagnostic accuracy and efficiency. However, the responsible use of AI algorithms is paramount. Ensuring that AI models are transparent, unbiased, and ethically sound is crucial to maintaining trust in healthcare systems. A study published in *JAMA Network Open* emphasized the importance of transparency in AI algorithms. Approximately 63% of surveyed individuals expressed concerns about the "black box" nature of AI decisions, indicating the need for clarity and accountability in AI-driven diagnostics. As the landscape of digital pathology evolves, legal frameworks come into play to ensure compliance and accountability [24]. Regulatory approvals from bodies such as the Food and Drug Administration (FDA) are pivotal in validating the safety and effectiveness of digital pathology solutions. These approvals signify a commitment to patient safety and clinical reliability. Data from the FDA's digital pathology approval process reveal the meticulous scrutiny applied to these innovations. In 2020, the FDA cleared its first WSI system for primary diagnostic use. This marked a significant step in recognizing the potential of digital pathology in clinical practice [25].

Alongside regulatory considerations, the question of liability looms. In the event of diagnostic discrepancies or errors, the allocation of responsibility becomes paramount. The convergence of human expertise and AI-driven insights introduces complexities in determining accountability [26].

Pioneering Progress with Ethical Vigilance and Legal Prudence

In the tapestry of progress that digital pathology weaves, ethical and legal considerations serve as threads that bind innovation with responsibility. Patient data privacy, consent, responsible AI use, regulatory approvals, and liability concerns form the ethical and legal scaffolding upon which this paradigm rests. In the backdrop of these considerations, digital pathology emerges as a transformative force that transcends geographical barriers, empowers diagnostics through AI-driven insights, and enhances patient care through more accurate diagnoses [27]. Remote consultations foster collaborations among experts worldwide, heralding a future where medical knowledge transcends borders. The promise of AI-assisted analysis holds the potential to minimize diagnostic errors and expedite treatment decisions. As AI algorithms evolve, they become indispensable allies in the pathologist's journey toward precision medicine [30].

## Conclusions

In conclusion, the ongoing trajectory of digital pathology calls for a harmonious collaboration between healthcare professionals and technology experts. The synergy between human expertise and technological innovation propels the field toward unparalleled heights. The ethical compass ensures that patient data is treated with the utmost care. At the same time, legal considerations provide a framework for accountability and safety. As digital pathology shapes the landscape of medical diagnostics, the promise of a future marked by ethical responsibility and pioneering progress stands as a testament to humanity's capacity to embrace innovation with wisdom and compassion.

## References

[1] Baxi V, Edwards R, Montalto M, Saha S (2022). Digital pathology and artificial intelligence in translational medicine and clinical practice. Mod Pathol.

[2] Bera K, Schalper KA, Rimm DL, Velcheti V, Madabhushi A (2019). Artificial intelligence in digital pathology - new tools for diagnosis and precision oncology. Nat Rev Clin Oncol.

[3] Ibrahim A, Gamble P, Jaroensri R, Abdelsamea MM, Mermel CH, Chen PC, Rakha EA (2020). Artificial intelligence in digital breast pathology: techniques and applications. Breast.

[4] Jiang Y, Yang M, Wang S, Li X, Sun Y (2020). Emerging role of deep learning-based artificial intelligence in tumor pathology. Cancer Commun (Lond).

[5] Calderaro J, Seraphin TP, Luedde T, Simon TG (2022). Artificial intelligence for the prevention and clinical management of hepatocellular carcinoma. J Hepatol.

[6] Shmatko A, Ghaffari Laleh N, Gerstung M, Kather JN (2022). Artificial intelligence in histopathology: enhancing cancer research and clinical oncology. Nat Cancer.

[7] Yousif M, van Diest PJ, Laurinavicius A (2022). Artificial intelligence applied to breast pathology. Virchows Arch.

[8] Nagarajan VD, Lee SL, Robertus JL, Nienaber CA, Trayanova NA, Ernst S (2021). Artificial intelligence in the diagnosis and management of arrhythmias. Eur Heart J.

[9] Mintz Y, Brodie R (2019). Introduction to artificial intelligence in medicine. Minim Invasive Ther Allied Technol.

[10] Niu PH, Zhao LL, Wu HL, Zhao DB, Chen YT (2020). Artificial intelligence in gastric cancer: application and future perspectives. World J Gastroenterol.

[11] Sharma P, Hassan C (2022). Artificial intelligence and deep learning for upper gastrointestinal neoplasia. Gastroenterology.

[12] Försch S, Klauschen F, Hufnagl P, Roth W (2021). Artificial intelligence in pathology. Dtsch Arztebl Int.

[13] Shen Z, Hu J, Wu H (2022). Global research trends and foci of artificial intelligence-based tumor pathology: a scientometric study. J Transl Med.

[14] Majumder A, Sen D (2021). Artificial intelligence in cancer diagnostics and therapy: current perspectives. Indian J Cancer.

[15] Wells A, Patel S, Lee JB, Motaparthi K (2021). Artificial intelligence in dermatopathology: diagnosis, education, and research. J Cutan Pathol.

[16] Hung KF, Ai QY, Leung YY, Yeung AW (2022). Potential and impact of artificial intelligence algorithms in dento-maxillofacial radiology. Clin Oral Investig.

[17] Hornung AL, Hornung CM, Mallow GM (2022). Artificial intelligence in spine care: current applications and future utility. Eur Spine J.

[18] Farris AB, Vizcarra J, Amgad M, Cooper LA, Gutman D, Hogan J (2021). Artificial intelligence and algorithmic computational pathology: an introduction with renal allograft examples. Histopathology.

[19] Lancellotti C, Cancian P, Savevski V, Kotha SR, Fraggetta F, Graziano P, Di Tommaso L (2021). Artificial intelligence & tissue biomarkers: advantages, risks and perspectives for pathology. Cells.

[20] Goldenberg SL, Nir G, Salcudean SE (2019). A new era: artificial intelligence and machine learning in prostate cancer. Nat Rev Urol.

[21] Xiang Y, Zhao L, Liu Z (2020). Implementation of artificial intelligence in medicine: Status analysis and development suggestions. Artif Intell Med.

[22] Tran NK, Albahra S, May L, Waldman S, Crabtree S, Bainbridge S, Rashidi H (2021). Evolving applications of artificial intelligence and machine learning in infectious diseases testing. Clin Chem.

[23] Yao K, Singh A, Sridhar K, Blau JL, Ohgami RS (2020). Artificial intelligence in pathology: a simple and practical guide. Adv Anat Pathol.

[24] Bang CS, Lee JJ, Baik GH (2020). Artificial intelligence for the prediction of helicobacter pylori infection in endoscopic images: systematic review and meta-analysis of diagnostic test accuracy. J Med Internet Res.

[25] Rakha EA, Toss M, Shiino S, Gamble P, Jaroensri R, Mermel CH, Chen PC (2021). Current and future applications of artificial intelligence in pathology: a clinical perspective. J Clin Pathol.

[26] Ting DS, Foo VH, Yang LW (2021). Artificial intelligence for anterior segment diseases: emerging applications in ophthalmology. Br J Ophthalmol.

[27] Feng C, Liu F (2023). Artificial intelligence in renal pathology: current status and future. Biomol Biomed.

[28] Miller DD, Brown EW (2018). Artificial intelligence in medical practice: the question to the answer?. Am J Med.

[29] Lin E, Fuda F, Luu HS, Cox AM, Fang F, Feng J, Chen M (2023). Digital pathology and artificial intelligence as the next chapter in diagnostic hematopathology. Semin Diagn Pathol.

[30] Chassagnon G, De Margerie-Mellon C, Vakalopoulou M, Marini R, Hoang-Thi TN, Revel MP, Soyer P (2023). Artificial intelligence in lung cancer: current applications and perspectives. Jpn J Radiol.

